# Priorities, opportunities, and challenges for integrating microorganisms into Earth system models for climate change prediction

**DOI:** 10.1128/mbio.00455-24

**Published:** 2024-03-25

**Authors:** J. T. Lennon, R. Z. Abramoff, S. D. Allison, R. M. Burckhardt, K. M. DeAngelis, J. P. Dunne, S. D. Frey, P. Friedlingstein, C. V. Hawkes, B. A. Hungate, S. Khurana, S. N. Kivlin, N. M. Levine, S. Manzoni, A. C. Martiny, J. B. H. Martiny, N. K. Nguyen, M. Rawat, D. Talmy, K. Todd-Brown, M. Vogt, W. R. Wieder, E. J. Zakem

**Affiliations:** 1 Department of Biology, Indiana University, Bloomington, Indiana, USA; 2 Lawrence Berkeley National Laboratory, Berkeley, California, USA; 3 Ronin Institute, Montclair, New Jersey, USA; 4 Department of Ecology and Evolutionary Biology, University of California Irvine, Irvine, California, USA; 5 Department of Earth System Science, University of California Irvine, Irvine, California, USA; 6 American Society for Microbiology, Washington, DC, USA; 7 Department of Microbiology, University of Massachusetts, Amherst, Massachusetts, USA; 8 NOAA/OAR Geophysical Fluid Dynamics Laboratory, Princeton, New Jersey, USA; 9 Department of Natural Resources and the Environment, University of New Hampshire, Durham, New Hampshire, USA; 10 College of Engineering, Mathematics, and Physical Sciences, University of Exeter, Exeter, United Kingdom; 11 Department of Plant and Microbial Biology, North Carolina State University, Raleigh, North Carolina, USA; 12 Department of Biological Sciences, Center for Ecosystem Science, Northern Arizona University, Flagstaff, Arizona, USA; 13 Department of Physical Geography, Bolin Centre for Climate Research, Stockholm University, Stockholm, Sweden; 14 Department of Ecology and Evolutionary Biology, University of Tennessee, Knoxville, Tennessee, USA; 15 Department of Biological Sciences, University of Southern California, Los Angeles, California, USA; 16 National Science Foundation, Washington, DC, USA; 17 Department of Microbiology, University of Tennessee, Knoxville, Tennessee, USA; 18 Department of Environmental Engineering Sciences, University of Florida, Gainesville, Florida, USA; 19 Institute for Biogeochemistry and Pollutant Dynamics, ETH Zürich, Zürich, Switzerland; 20 National Center for Atmospheric Research, Boulder, Colorado, USA; 21 Institute of Arctic and Alpine Research, University of Colorado, Boulder, Colorado, USA; 22 Department of Global Ecology, Carnegie Institution for Science, Stanford, California, USA; The University of Tennessee Knoxville, Knoxville, Tennessee, USA

**Keywords:** biogeochemistry, modeling, traits, climate change

## Abstract

Climate change jeopardizes human health, global biodiversity, and sustainability of the biosphere. To make reliable predictions about climate change, scientists use Earth system models (ESMs) that integrate physical, chemical, and biological processes occurring on land, the oceans, and the atmosphere. Although critical for catalyzing coupled biogeochemical processes, microorganisms have traditionally been left out of ESMs. Here, we generate a “top 10” list of priorities, opportunities, and challenges for the explicit integration of microorganisms into ESMs. We discuss the need for coarse-graining microbial information into functionally relevant categories, as well as the capacity for microorganisms to rapidly evolve in response to climate-change drivers. Microbiologists are uniquely positioned to collect novel and valuable information necessary for next-generation ESMs, but this requires data harmonization and transdisciplinary collaboration to effectively guide adaptation strategies and mitigation policy.

## OPINION/HYPOTHESIS

For more than a century, scientists have been developing models to understand and predict the complexity of Earth system dynamics (1). Earth system models (ESMs) are built from a collection of submodels that represent interactions among processes occurring on land, in the oceans, and in the atmosphere. ESMs are intended to capture emergent properties and feedbacks that operate at large scales, ranging from biomes to the full planetary system. However, there is continuous effort and ongoing debate concerning if, how, and when smaller-scale processes should be incorporated into ESMs.

**Fig 1:**
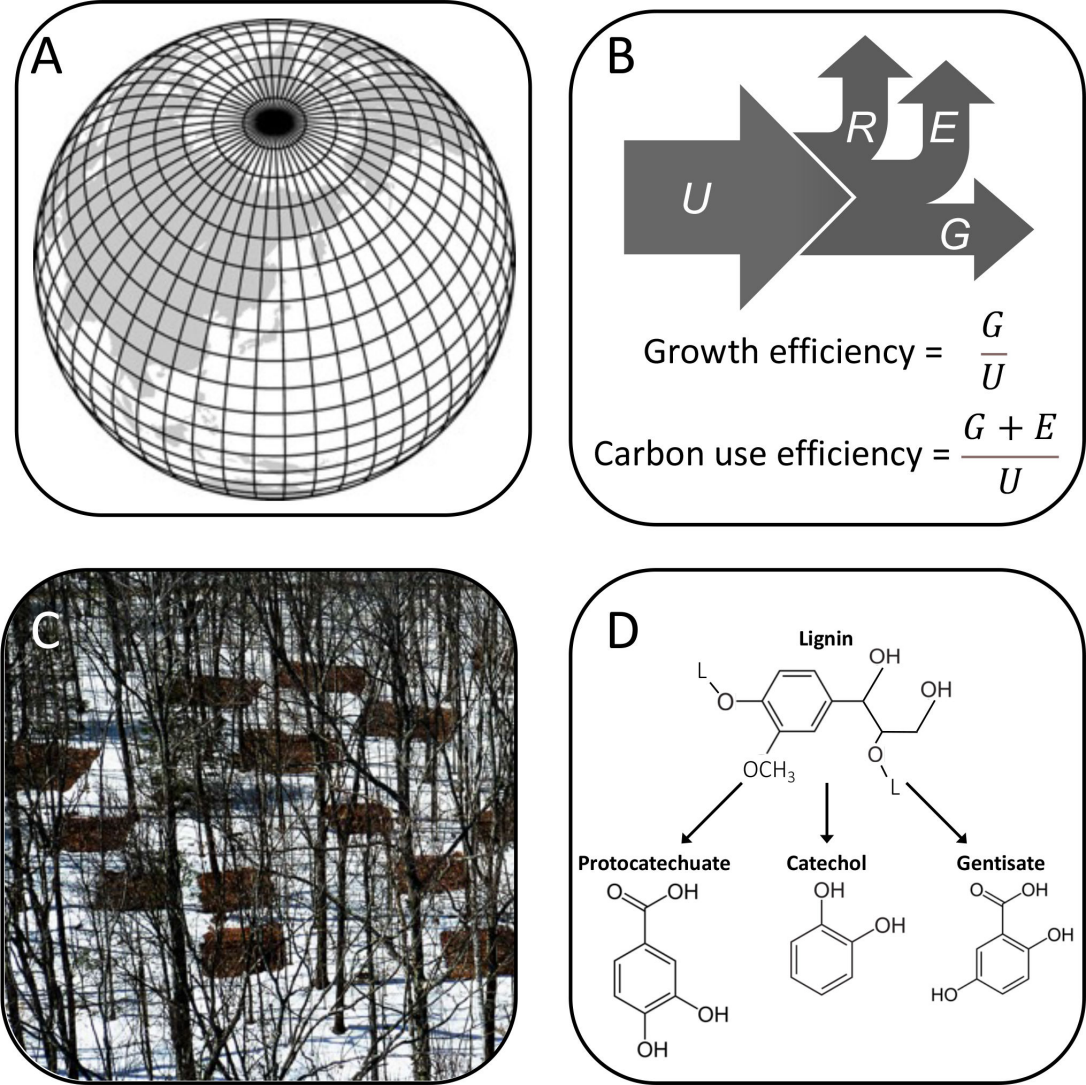
Microbes in models. (**A**) ESMs consist of submodels that represent physical, chemical, and biological interactions that control processes on land, in the ocean, and in the atmosphere with grid scales that are typically 100 km × 100 km, or approximately one degree of latitude and longitude. (Map image from reference 4.) (**B**) A microbially informed ESM might contain equations that describe how cells process carbon (*C*) which includes estimates for uptake (*U*), respiration (*R*), exudation (*E*), and growth (*G*), which can also include categorical or continuous traits, such as enzyme kinetics or temperature sensitivities that act as rate modifiers with effects on ecosystem functioning that can generate potential feedback. (**C**) Equations in ESM need to be parameterized, ideally with information collected from experiments and comparative studies that capture key environmental drivers associated with climate change on relevant temporal and spatial scales. Pictured here in the winter are plots from a long-term warming experiment at the Harvard Forest in Massachusetts, USA (copyright Audrey Barker Plotkin), where microbial data have been critical for understanding soil carbon feedback to climate systems (5). (**D**) The incorporation of microbes into ESMs can provide mechanistic insight into how traits and functional groups affect biogeochemical processes. For example, lignin is a complex polymer derived from the cell wall of plants that is important for understanding soil carbon dynamics. Comprising cross-linked lignols (L), its degradation is initiated by microbial depolymerization followed by funneling pathways that catabolize different aromatic compounds depending on distinct classes of fungal and bacterial enzymes (6), which can be affected by environmental conditions that are associated with climate change.

First and foremost, ESMs must capture the physics and chemistry of the planet. Thus, the models encode thermodynamics, turbulence, fluid dynamics, radiation, and the multiphasic transitions of water. These features are combined with biogeochemical processes and are represented on a three-dimensional grid (2). Once an ESM is developed, it needs to be validated with respect to historical trends in data that might span seasonal to millennial time scales before being used to project the future effects of natural and anthropogenic changes (Fig. 1) (3).

With advances in computational power, ESMs have become more complex over the past few decades. Many biological processes are now represented in ESMs. On land, this includes the modeling of energy and mass exchanges mediated by vegetation, such as evapotranspiration and photosynthesis, and the dynamics of plant-soil carbon stocks (7). In the oceans, ESMs typically include carbonate chemistry, plankton interactions, nutrient limitation, and processes that export particulate organic carbon to the deep sea (8). These efforts have confirmed that biological agents interact with and mediate many chemical and physical processes, and that they generate feedbacks that can influence the biosphere under current and future climate-change scenarios.

ESMs have the potential to make better predictions with a more explicit representation of key microbial processes (9, 10). As the most abundant and diverse forms of life on Earth, microorganisms catalyze biogeochemical processes that affect the storage and transformation of carbon and nitrogen at the global scale. In addition to controlling the turnover of other important elements, microorganisms produce and consume trace gases that directly contribute to climate forcing. Although it is widely understood that microorganisms play a significant role in global biogeochemistry, only recently have some attempts been made to include them into ESMs. However, consensus is currently lacking on how best to accomplish this in a way that improves model accuracy under different climate-change scenarios. In this Opinion piece, we identify priorities, challenges, and opportunities with the goal of facilitating the integration of microbial data into effective modeling frameworks.

## TOP 10 PRIORITIES, OPPORTUNITIES, AND CHALLENGES

### Determine when explicit microbial representation is required

A priority for Earth system science is to determine how sensitive model predictions are to the inclusion of microorganisms. Many existing ESMs do a reasonably good job of representing large-scale ecosystem dynamics without explicitly encoding microorganisms. This raises questions about whether or not incorporating more microbial information will add value to ESMs. As an example, consider rice paddies, which cover more than 150 million hectares of Earth’s surface, mostly in South Asia (11). During the wet seasons, pore spaces in paddy soils become saturated leading to oxygen depletion and a concomitant increase in CH_4_ efflux to the atmosphere. Methods exist to predict these greenhouse gas emissions using satellite data and meteorological information (12) without extensive measurements of methanogen and methanotroph dynamics that would be needed to parametrize a microbially explicit ESM. In this scenario, an argument could be made that explicit microbial representation may not be critical for understanding how hydrology and agricultural practices in one region of the world influence CH_4_ dynamics so long as functions can be adequately represented. Therefore, a major challenge is to identify what types of information can better inform ESMs and reconcile explicit process representation at the microbial scale with ecosystem scale functional responses.

### Establish the optimal degree of model complexity

While existing models may perform reasonably well under some scenarios, there are compelling reasons to build ESMs that explicitly represent microorganisms. Their omission is at odds with the knowledge that microbes play a key role in virtually all food webs and ecosystems on the planet. While their contributions to Earth system dynamics can sometimes be implicitly captured in ESMs, microorganisms may create unexpected feedbacks owing to non-linear and interactive responses to multiple climate-change drivers. Furthermore, while existing models may be adequate for capturing past or current Earth system dynamics, it is unclear whether they will perform well in the future under conditions for which models are not sufficiently parameterized. In fact, soil models that incorporate aspects of microbial physiology (i.e., growth efficiency) have been shown to do a better job of predicting decomposition and global-scale carbon storage than models that represent microbial activities only implicitly (13) (Fig. 1). Likewise, the representation of different growth rates and thermal traits for microbial phototrophs in marine ecosystems has consequences not only for species diversity and biogeography but also rates of primary production and the export of silica from surface waters of the global ocean (14). However, there are costs to adding too many microbial features to ESMs. Already, most ESMs are complex, computationally intensive, and have an excess of free parameters. Therefore, when adding new microbial variables, it is a priority and challenge to carefully consider parameterization and the degrees of freedom that are introduced, which can otherwise lead to unconstrained output and more variability in model projections (15). Coordination among research teams is needed to determine the optimal balance between additional microbial information and model complexity.

### Identify microbial functional groups for inclusion in ESMs

Microbial communities are extremely complex. When building a microbially explicit ESM, it would not be practical or desirable to resolve all species or metabolic pathways for the billions of individuals that are commonly found in a gram of soil or a liter of seawater. Some degree of coarse-graining is required. This can be achieved by grouping organisms together based on their functional traits, which are the morphological, physiological, and behavioral characteristics that influence the performance of an organism under a set of environmental conditions (16). For decades, a functional-group framework has been used to simplify biological complexity in ESMs. On land, modelers may represent different types of biomes based on plant leaf properties (deciduous vs evergreen) or photosynthetic pathways (C3 vs C4). In the oceans, some models capture important trade-offs in organismal function such as the ability to fix atmosphere nitrogen, sequester silica, or produce aerosols (e.g., dimethyl sulfide) that can affect cloud condensation (17). Similar trait-based approaches could be extended to model other microbial functions in different ecosystems (18). Taxa can be grouped according to their metabolism (e.g., sulfate reducers, iron oxidizers, anaerobic phototrophs), resource use, stress response strategies, or capacity to emit greenhouse gasses (19). In a less categorical fashion, microbial activities can be expressed as a continuous function of climatically relevant environmental drivers, such as temperature, oxygen, or moisture, which is akin to the rate modifiers that are already implemented into ESMs. However, the currently used modifiers are derived from empirical observations that do not account for microbial responses (e.g., acclimation or adaptation) to changing environments. While there are opportunities to leverage existing trait-based approaches and data, additional work is needed to further refine the relevant microbial traits and functional groups to include in ESMs.

### Reconcile the spatial scales of microorganisms and the Earth system

Integrating spatial processes is a major challenge when attempting to incorporate microorganisms into ESMs. At the scale of a typical microbial cell (1 µm^2^), important processes take place, including the uptake of growth-limiting resources; the diffusion of substrates, enzymes, and signaling molecules to and from neighboring cells; and stochastic encounters with infectious viruses. At large scales, other processes such as dispersal are critical for understanding the biogeographic distribution of microbial functional groups. Therefore, the scale-dependence of processes and patterns must be considered when incorporating microorganisms into ESMs, which are often resolved at 100 km^2^ grid sizes or larger. For example, the mathematical structure used to model the non-linear kinetics of soil respiration changes when moving from small to large scales owing to environmental heterogeneity (20). Fortunately, there are opportunities to resolve these problems. For example, hierarchical approaches have been used by biologists, physicists, and engineers for dealing with the challenges that are associated with spatial scaling, some of which have already been implemented into ESMs (21).

### Reconcile the temporal scales of microorganisms and the Earth system

Temporal scale is also a challenge when incorporating microorganisms into ESMs. Many microbial processes—including gene regulation, substrate diffusion, and even cell division times—occur rapidly, on the order of seconds to minutes. Although measurements of such phenomena represent snapshots in time, some of these processes could have carry-over effects that extend for longer periods of time. Moreover, microbial metabolism is energetically or nutritionally constrained for vast portions of the globe and this can greatly prolong the persistence and lifespan of cells with consequences for biomass turnover, a process that is highly relevant for modeling ecosystem dynamics (22). A priority for future ESMs is to capture recurring patterns of microbial change associated with seasonality or succession. More information is needed to accurately describe the time scale at which microbial communities respond to and recover from stressors associated with climate change. Most models assume that microbes can instantaneously acclimate to fluctuations in their environment, but lags and memory may contribute to their responsiveness to perturbations (23). Such information is not only important for making model predictions but also for management decisions aimed at mitigating the effects of climate change. Last, it is important to note that microbial responses may depend on the temporal dynamics of the stressor in question, with dynamics differing from episodic extreme events to chronic long-term changes in environmental conditions (24). Fortunately, components of existing ESMs are already capable of updating chemical, physical, and biological processes on relatively short time scales (e.g., minutes to hours), which can, in principle, accommodate the dynamics of many microbial phenomena.

### Account for rapid evolutionary change

In nature, populations can evolve when confronted by novel environmental conditions, but this is generally not represented in most ESMs. Compared to other taxa, microorganisms may be somewhat unique in their ability to adapt to climate change owing to large effective population sizes, short generation times, and the capacity to horizontally transfer genetic material among distantly related lineages. While most examples of rapid evolution come from studies where microorganisms have been maintained under highly controlled laboratory conditions, there is growing evidence that genetic changes arise in wild populations when they are challenged by climate-change drivers (25). It remains to be determined whether these evolutionary changes demonstrably affect ecosystem functioning. If so, then scientists will need to grapple with how best to mathematically embed adaptive and nonadaptive evolutionary dynamics into ESM structures. While this may represent a computational challenge, it is also an opportunity for using modeling platforms to test hypotheses about the importance of evolutionary processes and their feedback at the global scale. Efforts are already underway to tackle such problems, for example, using adaptive dynamics modeling of phytoplankton at spatial and temporal scales that are potentially relevant to ESM predictions (26).

### Resolve microbial processes at the land-water interface

Climate change is expected to have strong effects at the land-water interface. In addition to its devastating socioeconomic consequences, sea-level rise will alter the biology of coastal ecosystems. For example, saltwater intrusion modifies the composition and productivity of plant communities, as well as the biogeochemistry of wetlands and upland soils (6). In the opposite direction, large quantities of fertilizers, pesticides, and plastics are exported from terrestrial to aquatic ecosystems. Collectively, these fluxes at the land-water interface may create microbially mediated feedbacks that are relevant for Earth system dynamics. However, modelers and microbiologists often treat land and water as separate entities, which overlooks important linkages between these systems. Thus, a priority is to further develop microbially explicit ESMs that also account for cross-system fluxes of material and energy. Such models will foster intellectual exchange between scientific communities, while also generating basic knowledge that can help address the climate crisis.

### Collect the right information

Often, climate-change models are in need of more basic biological data that directly relates to Earth system processes. For example, estimates of microbial abundance, biomass, and productivity at higher spatial and temporal resolutions would be most relevant for ESMs. While these methods have been well-vetted for decades, we are in an era where cutting-edge molecular technologies are greatly advancing the field of microbiology. Large volumes of metagenomic, metatranscriptomic, metaproteomic, and metabolomic data are being generated from diverse habitats across the globe. While these efforts have provided novel insight into the structure and function of microbial communities, the raw information is not particularly useful for modelers whose primary aim is to predict Earth system dynamics (27, 28). Microbiologists will often attempt to reduce the dimensionality of large omics data sets using multivariate statistics or network analyses. The products from these commonly used approaches cannot easily be translated into quantities that inform ESMs, but there are opportunities to generate composite metrics or use omics data in other creative ways to better model ecosystem dynamics (29). For example, oxidative and hydrolytic enzymes estimated from gene abundance data have been incorporated into decomposition models to predict soil respiration (30). In addition, metagenomic approaches have been used to project nutrient stress in the global ocean (31). Ultimately, ESM development will be advanced by microbiologists, ecologists, and modelers working together to design experimental and comparative approaches that synthesize data from large-scale climate-change studies (32).

### Promote data harmonization

The data needed for developing new ESMs come from many sources and are collected by researchers spanning a range of disciplines. Data harmonization can facilitate the integration and efficient use of such information. Ontologies are needed so that researchers have a shared list of categories and concepts with clearly defined relationships (33). Such structures will help ensure that data are obtained and processed in a standardized and compatible fashion for the construction and sharing of databases that are used in ESM development. It is essential that the microbial information in these databases be accompanied by metadata (34). Geographic coordinates, temperature, redox potential, and other environmental data are priorities for modelers whose goal may be to relate microbial processes to state variables and transformations in an ESM. Ultimately, data harmonization requires a data-centered community of practice that will reinforce transdisciplinary collaboration and facilitate the development of microbially informed ESMS.

### Develop models to advance microbial knowledge

From an Earth system modeling perspective, microbes are important to consider when they create feedbacks and modify predictions for a given climate-change scenario. Regardless of the magnitude of their effect, there is still value in attempting to include microorganisms in ESMs. In addition to generating synthetic knowledge of the microbial biosphere and its role in biogeochemical cycles, such efforts will help with the quantitative and cross-disciplinary training of early career scientists, the building of research networks, and the organization of complex data. Even if the effects are most pronounced at local or regional scales, updated models will yield new insight into how climate change affects the distribution, abundance, and functionality of microbial life, which is important for the stability of managed and natural ecosystems and the services that they provide.

## CONCLUSION

While there are many challenges and considerations, the inclusion of microbial information into ESMs provides opportunities for developing more realistic and useful climate-change predictions. From the opposite direction, ESMs have the potential to identify microbial processes and relationships that generate model uncertainty, which could inspire and guide the pursuit of biological discovery and knowledge. To attain these goals, greater transdisciplinary research coordination and communication are needed (35), which can be facilitated with new working groups, restructured conferences, and the initiation of multi-institutional centers supported by universities, industry, national laboratories, and federal funding agencies. Collaborative teams made up of climate scientists and modelers will be well-positioned to design and execute research projects that will generate suitable types of data needed to construct microbially informed ESMs that can guide future policy and climate-change mitigation strategies.
